# Transposon Insertion Mutagenesis in Mice for Modeling Human Cancers: Critical Insights Gained and New Opportunities

**DOI:** 10.3390/ijms21031172

**Published:** 2020-02-10

**Authors:** Pauline J. Beckmann, David A. Largaespada

**Affiliations:** 1 Department of Pediatrics, University of Minnesota, Minneapolis, MN 55455, USA; pjjackso@umn.edu; 2 Masonic Cancer Center, University of Minnesota, Minneapolis, MN 55455, USA; 3 Department of Genetics, Cell Biology and Development, University of Minnesota, Minneapolis, MN 55455, USA; 4 Center for Genome Engineering, University of Minnesota, Minneapolis, MN 55455, USA

**Keywords:** animal modeling, cancer, transposon screen

## Abstract

Transposon mutagenesis has been used to model many types of human cancer in mice, leading to the discovery of novel cancer genes and insights into the mechanism of tumorigenesis. For this review, we identified over twenty types of human cancer that have been modeled in the mouse using *Sleeping Beauty* and *piggyBac* transposon insertion mutagenesis. We examine several specific biological insights that have been gained and describe opportunities for continued research. Specifically, we review studies with a focus on understanding metastasis, therapy resistance, and tumor cell of origin. Additionally, we propose further uses of transposon-based models to identify rarely mutated driver genes across many cancers, understand additional mechanisms of drug resistance and metastasis, and define personalized therapies for cancer patients with obesity as a comorbidity.

## 1. Transposon Basics

Until the mid of 1900’s, DNA was widely considered to be a highly stable, orderly macromolecule neatly organized into chromosomes. Barbara McClintock challenged this paradigm in 1950 when she published her studies on the first transposable elements, *Ac* and *Ds*, which she discovered in maize [1]. She found that these transposable elements, or transposons, could cause large genetic changes and reversibly alter gene expression. Transposons have been classified based on their mechanism of movement throughout the genome (transposition). Class I is made up of retrotransposons which mobilize through an RNA intermediate-based “copy-and-paste” mechanism. This review will focus on class II elements, which use a DNA-mediated “cut-and-paste” mode of transposition. In nature, transposons encode an enzyme to direct their transposition called a transposase, and transposase recognition sequences on both ends (terminal inverted repeats (TIRs)), which direct transposase binding and mobilization of the transposon. For use in a laboratory setting, the transposon and transposase can be physically separated, with the transposase supplied in *trans*. This allows the transposon to encode alternative DNA sequences and for the system to be more intricately regulated. Transposon insertion is a mutagenic process and can result in both gain and loss of function mutations.

Transposition technology can be used in both “forward” and “reverse” genetic studies. Reverse genetics involves targeting a specific gene of interest to facilitate gain or loss of function studies. For example, knocking out or overexpressing a putative oncogene in a relevant cell line and analyzing the resultant phenotypic changes. These studies are quite useful for validation and functional analysis of single genes but are limited in their scope. Forward genetic studies obtain a phenotype through mutagenesis on a genome-wide scale, allowing the study of many genes and pathways simultaneously. For example, chemical mutagens, ionizing radiation, or transposition can be used to create a desired phenotype (i.e., change in leaf structure or tumor formation), and then mapping of the associated genetic changes will give insight into what genes or gene sets are involved in the phenotype under study.

Transposons have been used to study gene function successfully in many organisms, including yeast, plants, invertebrates, and vertebrates. For example, the prokaryotic bacteriophage *Mu* transposition complex has been used to disrupt gene expression in yeast, mouse, and human cells [2]. The maize DNA transposons *Ac/Ds*, *En/Spm*, and *Mu* have been used in maize, rice, tomato, and *Arabidopsis* [3,4,5,6,7]. The *Drosophila mauritiana* transposon *Mos1* has been used successfully in several forward genetic screens in *Caenorhabditis elegans* to identify important genes in a variety of biological processes [8,9,10,11]. *P* element transposons and transposable elements with diverse insertional specificities including *Tol2*, *piggyBac* (*PB*), and *Minos* have been instrumental to our current understanding of the *Drosophila melanogaster* genome [12,13,14,15]. *Tol2* (isolated from medaka fish) and insect-derived *PB* and *Minos* have also been used in mutagenesis in vertebrates such as the mouse and zebrafish [16,17,18]. *Sleeping Beauty* (*SB*) is derived from elements cloned from salmonid fish and has been widely used in insertional mutagenesis screens in mice [19,20,21,22,23,24,25,26,27,28,29] and shown to be active in other vertebrates including cultured cell lines, rats, zebrafish, and *Xenopus* [19,30,31,32].

The main practical differences between transposable elements include cargo capacity, integration site preference, and the rate of “local hopping.” Cargo capacity varies greatly among transposable elements; this is an important factor to consider, particularly for delivery of complex genetic cargos or longer genes. Transposition frequency of Tc1/*mariner* family members, including *SB* and *Minos*, decreases with increasing transposon length [33,34,35], although *SB* has shown to be able to deliver very large BAC constructs (>60 kb) [36] and has been modified to handle large sequences with more efficiency (>10 kb) [37]. *PB* and *Tol2* are more tolerant of increasing transposon size, making them a preferred choice for larger sequences [16,38]. Integration site preference is also important to consider when choosing the appropriate transposon vector. For use in mutagenesis, it is preferable to use a transposon system with a propensity to land within genes, like *PB*, to increase the chance of changing gene expression [39]. On the other hand, a nearly random mutagenesis system is likely to have less bias for a subset of genes. For use in a gene therapy setting, systems without a proclivity for transcriptional units like *SB*, are superior [40]. Some transposons display a sequence preference for integration, with Tc1/*mariner* elements (*SB*, *Frog Prince*, *Minos*, and *Hsmar1*) integrating into a TA dinucleotide sequence and *PB* targeting a “TTAA” sequence. In the case of *SB*, DNA structure and bendability are the primary predictive factor for integration and compared to other transposon systems, *SB* integration is affected little by gene content or other genomic features, making it an ideal tool for random mutagenesis [41]. Finally, local hopping, or a preference for transposons to land into cis-linked sites in close proximity of the donor locus, plays a significant role in the saturation efficiency during a mutagenesis experiment. *PB* and *SB* both exhibit local hopping, although *PB*-mediated local hopping is less pronounced [39,42]. Local hopping may be advantageous for a particular experiment, for example if saturation of a specific chromosome is of interest. If not, it can be circumvented by use of multiple transposon locations and/or taken into account during the analysis of the mutation data generated.

In comparison to other methods of identifying genetic drivers of cancer such as CRISPR/Cas9 or retroviruses, transposon insertion mutagenesis has its advantages and disadvantages (Table 1). While both CRISPR/Cas9 and transposon systems can cover the entire genome, transposon screens carry a slight bias related to local hopping and insertion preference that can be eliminated with careful guide RNA library design. However, in the context of in vivo models of cancer, CRISPR/Cas9 is hardly comparable to the utility of transposon mutagenesis. While CRISPR/Cas9 can be used to create loss and gain of function mutations, genome wide screens are done in such a way that each cell suffers a single mutation. Transposon mutagenesis in vivo allows for the accumulation of multiple, independent mutations that can cooperate to cause a phenotype. Therefore, transposon mutagenesis more accurately reflects the complexity of human cancer, which evolves in a stepwise manner. More recently these technologies have been combined by using a transposase (either PB or SB) to deliver single guide RNAs (sgRNAs) and Cas9 into mice in a reverse genetic approach [43,44]. Weber et al. delivered SB transposase and a transposon containing many sgRNA and *Cas9* sequences flanked by SB recognition sequences by tail vein injection resulting in the formation of hepatocellular carcinoma and intrahepatic cholangiocarcinoma [43]. This combination allowed delivery of multiple sgRNAs simultaneously and more high-throughput screening. Slow transforming retroviruses have been used to identify important drivers of mouse lymphoma (MuLV) and mammary tumors (MMTV) [45,46], however the application of these viruses is limited due to their cellular tropisms. The main advantage of transposon-based mutagenesis systems to retroviral screens is their tissue flexibility and the modifiable nature of the components, allowing tumorigenesis in nonlymphoid and non-mammary tissues.

## 2. Transposons to Model Human Cancer in Mice

**Figure 1:**
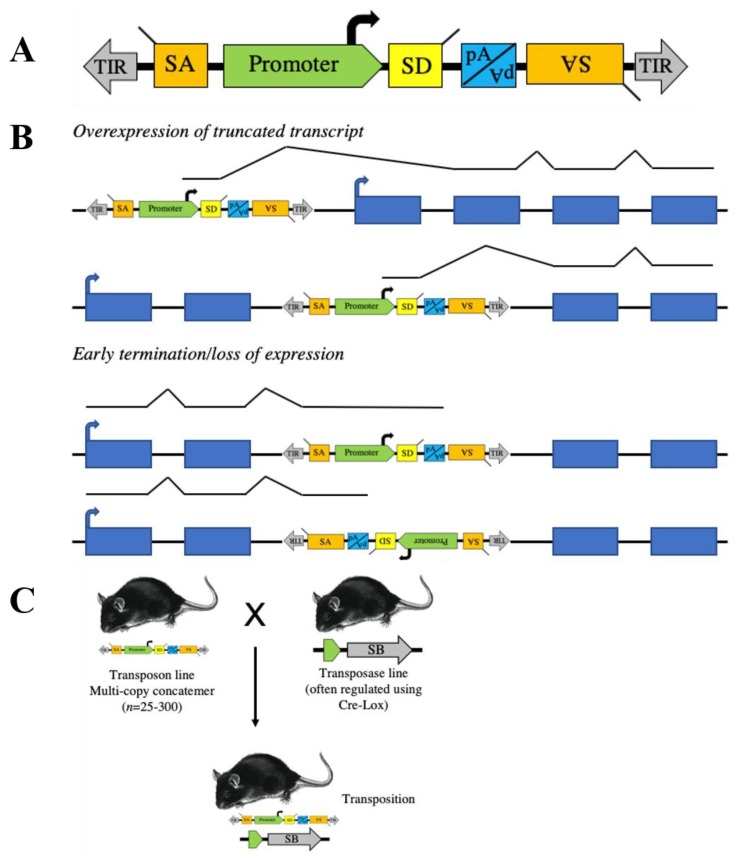
*Sleeping Beauty* (SB) transposons can be designed to randomly induce somatic cell gain and loss of function mutations. (**A**) Structure of a proto-typical transposon vector for somatic cell or cell line mutagenesis studies. A strong promoter followed by an exon with a splice donor (SD) is present to activate transcription of downstream exons. Splice acceptors (SA) and a bi-directional polyadenylation site (pA) are included to disrupt gene expression. (**B**) In mutagenized cells, transposons can activate endogenous proto-oncogenes or disrupt endogenous tumor suppressor genes depending on where insertion occurs and in what orientation. (**C**) Transposon transgenic mice are usually produced by standard pronuclear injection resulting in the generation of lines with multicopy concatomers. These are crossed to mice expressing the transposase to generate mice with somatic cell transposition.

Transposase systems, mainly *SB*, have been used to model and identify genetic drivers in many types of human cancer (Table 2). For use in forward genetic screens, the *SB* transposon and transposase have been modified to achieve sufficient mutagenesis to drive tumor formation (Figure 1A). The first transposons used, *T2/Onc* and *T2/Onc2*, use the murine stem cell virus long terminal repeat (MSCV-LTR) promoter followed by a splice donor (SD) sequence to drive gene expression and bidirectional splice acceptors (SA) and polyadenylation signal (pA) to terminate gene transcription and ablate expression [20,21]. This allows *SB*-mediated transposon insertion mutagenesis to identify both oncogene and tumor suppressor gene candidates (Figure 1B). An optimized *SB* transposase sequence (*SB11*) was knocked into the *Gt(ROSA)26Sor* locus, facilitating ubiquitous expression [21,34]. By crossing the *R26-SB11* mouse with mice carrying either *T2/Onc* or *T2/Onc2*, researchers were able to induce leukemia in mice (Figure 1C) [22]. Subsequently, a conditional *SB* mouse was created (*R26-lsl-SB11*), allowing tissue and temporal-specific transposition and modeling of very specific cancers [23]. For example, we used *Nestin*-driven *Cre recombinase* to drive *SB* expression solely in the developing central nervous system and to identify novel genetic drivers of childhood brain tumors [28]. The expression profiles of many of the Cre strains described in Table 1 have been characterized by The Jackson Laboratory [47]. While transposon-mediated mutagenesis screens have taught us a great deal about cancer development over the last two decades, we would like to focus on a few studies and overall lessons learned.

## 3. Cell of Origin

*SB* mutagenesis has been used to test the impact of the cell of origin and stage of differentiation on transformation potential. Berquam-Vrieze et al. initiated transposition at increasingly differentiated stages in T-cell development using Cre-inducible *SB* and 3 different *Cre* transgenes [78]. *Vav*-*iCre*, *Lck-Cre*, and *CD4-Cre* induce Cre expression in hematopoietic stem cells, immature T-cells without CD4 or CD8 expression, or late-stage T-cells expressing both CD4 and CD8, respectively. The authors found that *Vav-iCre* mice had a significantly shorter survival time, indicating hematopoietic stem cells are a more permissive cell for leukemia induction than more differentiated populations. In agreement with this, they found that there was an increased average number of driver insertion mutations per leukemia clone with increased differentiation. In other words, it took more “genetic hits” to transform a more differentiated cell of origin. This concept that transformation potential is lost with differentiation has been shown in other models, including intestinal cancer and medulloblastoma [107,108]. When Berquam-Vrieze et al. compared genetic drivers in tumors generated with the three Cre transgenes, they found significantly different gene profiles for each differentiation stage, suggesting that the biology of each cell of origin greatly affects the genetics of tumor development. Interestingly, Berquam-Vrieze and colleagues compared subsets of *SB*-induced mouse lymphoma and found that the *CD4-Cre* (most differentiated cell of origin) lymphoma matched the expression patterns of human ETP-ALL, a subtype of T-ALL defined by expression patterns of early T-cell precursors. This was unexpected, as this was the most differentiated cell of origin in the study. Therefore, this study sheds light on the potential cell of origin for human ETP-ALL, suggesting it may be a more differentiated T cell that regains expression patterns of earlier T-cell progenitors, rather than an undifferentiated more stem-like cell.

## 4. Identification of Rare Events

One challenge in human cancer genetics has been identifying rarely mutated driver mutations, including both tumor suppressors and proto-oncogenes. This has sometimes been referred to as the “long tail” problem, reflecting the large number of genes, that are altered in a relatively small percentage of cancer cases. Many such genes exist in a “gray area” and it cannot easily be determined if their alteration is selected for in human cancer development. Sequencing of many human cancer cases will be required to determine if their alteration is statistically significant [109]. Transposon-based forward genetic screens can provide contributing circumstantial data that such candidates may be driver alterations in cancer. Each screen that is completed reports a list of a few frequently mutated candidate genes and many more infrequently mutated candidates. When multiple screens are combined and analyzed together, infrequently altered drivers become more visible across many cancers. For example, *Rreb1* is a tumor suppressor gene that has been identified as a candidate driver in a low number of many tumor types, including intestinal and pancreatic cancer and B-cell lymphoma [24,59,85]. Another example is *Foxr2*. Transposon mutagenesis studies identified *Foxr2* as a strong candidate driver of malignant peripheral nerve sheath tumors (MPNST), osteosarcoma, and medulloblastoma [28,29,48,96]. Interestingly, human *FOXR2* is amplified and overexpressed in a subset of human MPNST and activated by translocation or amplification in a subset of human embryonal tumors of the central nervous system [29,110]. Various other studies indicate that *FOXR2* high level expression is a feature of a subset of many tumor types, where it is likely a driver mechanism [111,112,113]. 

Based on the nature of how transposon-based screens work, they are uniquely able to identify proto-oncogenes activated by creation of fusion transcripts. We queried the Candidate Cancer Gene Database of *SB* screen-derived cancer gene candidates for those with recurrent fusion transcripts among the TCGA [114]. Indeed, many of the *SB*-predicted oncogenes are activated by translocations similar to those described for *FOXR2*. This includes known proto-oncogenes like *ERG* and *RAF1*, but many more novel proto-oncogene candidates including *AMBRA1* and *RALY*. *AMBRA1* is a regulator of autophagy and has been shown to affect drug resistance in several cancers [115,116,117]. *RALY* is an RNA-binding protein implicated in metastasis and associated with poor prognosis in breast and colorectal cancer and hepatocellular carcinoma [118,119,120]. More analysis to identify novel fusion transcripts at the RNA level could identify more novel, poorly understood drivers that have been missed through traditional analysis but may have meaningful implications.

Thus, transposon-based screens pooled together can be used to identify and prioritize novel human oncogenes activated in a rare subset of many cancers. This is relevant for new clinical trial designs, called “basket trials,” in which a single drug is tested in a variety of tumor types with a specific genetic alteration. For example, chromosomal fusion events involving the carboxy-terminal kinase domain of *TRK* (tropomysosin receptor kinase) have been identified in many cancers and shown to drive constitutively active, ligand-independent signaling which results in tumorigenesis regardless of tissue origin [121,122,123,124,125,126]. Larotrectinib is a potent and selective inhibitor of TRK proteins and has shown a durable antitumor effect in patients with *TRK* fusions regardless of patient age or tumor type [127].

## 5. Drivers of Metastasis

Two screens, one in medulloblastoma and one in osteosarcoma, have sought to address the questions of clonality and drivers of metastasis [48,99]. These studies found that when drivers of primary and metastatic tumors were compared, there was varying degrees of overlap. In the case of osteosarcoma, there were rare instances where multiple metastatic tumors within the same mouse were significantly different from each other [48]. In the case of medulloblastoma, dissemination from the primary tumor likely occurred early in the tumor development, potentially in multiple “seeding” events or in an on-going fashion [99]. These findings indicate that metastatic cancer develops in a rare subclone, perhaps early after tumor development, and that using targeted therapies based on the drivers in the primary tumor will not be enough to eliminate metastatic tumors as other driver alterations may have taken over a primary role in tumor maintenance. Additionally, several strong candidate drivers of metastasis were identified in these papers, including alterations in *Pten*, *Gsk3b*, *Snap23,* and *Raf1* in osteosarcoma [48]. Loss of *PTEN* has since been identified as a marker of poor clinical prognosis and lung metastasis in osteosarcoma [128].

**Figure 2:**
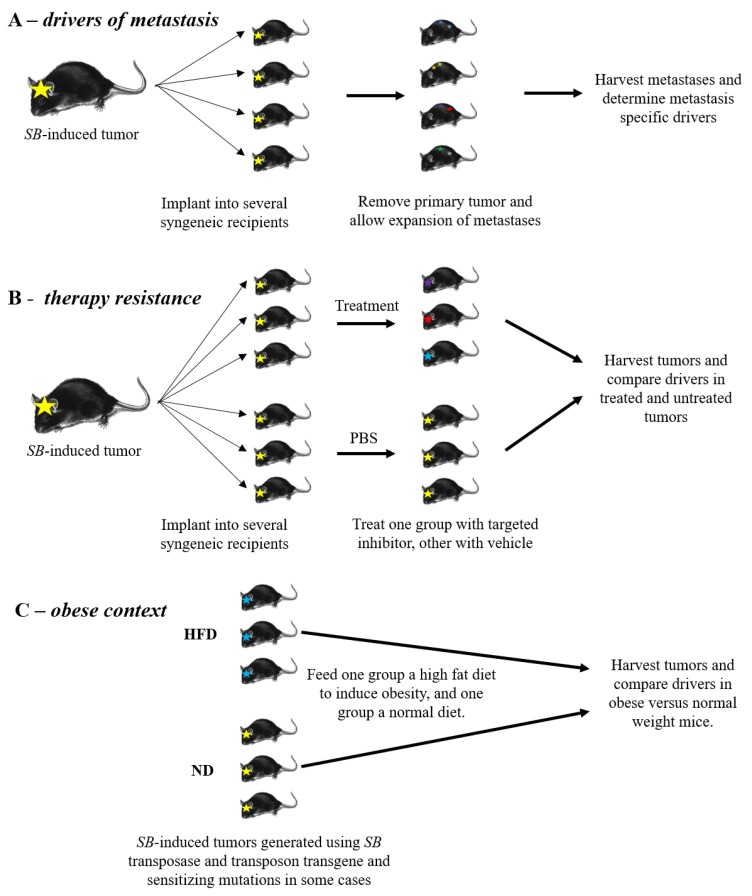
Future experiments for transposon insertional mutagenesis screens. (**A**,**B**) To identify drivers, induce tumor formation with insertional mutagenesis and implant tumors orthotopically into syngeneic recipients. (**A**) The primary tumor is expanded and removed before endpoint, allowing the metastatic lesions to expand and be harvested for driver analysis. (**B**) Implanted tumors are treated with a targeted inhibitor or left untreated and their drivers compared. (**C**) Mice undergoing mutagenesis are fed a normal or high fat diet. At endpoint all tumors are harvested and their drivers compared.

We believe that new transposon-based screens could be designed to better facilitate identification of metastatic drivers (Figure 2A). For practical purposes, a small screen can be done to produce a small number of primary tumors in mice expressing transposase, harboring a mutagenic transposon array, and any predisposing background mutations of interest. Primary tumors can then be removed and transplanted as allografts into multiple recipient mice. Ideally the tumors would be implanted orthotopically, and the primary tumor would be removed at a pre-defined size, allowing the metastases to expand. The timing of primary tumor removal will need to be optimized for every cancer type and experimental condition to balance death caused by the primary tumor and leaving the tumor in long enough for metastasis to occur. The metastatic clones can then be harvested and their genetic drivers identified in a much more expedited fashion compared to undergoing a full screen. The drivers identified in metastases can be compared to each other and the primary tumor to identify genes involved in metastasis and to provide knowledge on the clonality of the metastases in the cancer being studied. It may also be possible to provide adjuvant or neoadjuvant chemotherapy to better mimic the selective pressures that human metastases have undergone upon disease recurrence.

## 6. Therapy Resistance

Transposon-based mutagenesis in the presence of a targeted therapy offers a powerful tool for understanding genetic pathways to therapy resistance in cancer, which is a major problem in the quest for durable cures. For example, the *BRAF*^V600E^ mutation is present in approximately half of human melanoma, resulting in hyperactivation of the MAPK pathway [129]. While targeted therapy using vemurafenib was initially promising, tumors eventually recurred showing re-activation of the MAPK pathway [130]. Mann et al. performed *SB* mutagenesis in a *BRAF^V600E^*-driven mouse melanoma and identified many candidate cooperating genetic alterations [75]. Using a similar screening strategy, but including vemurafenib treatment of one cohort, Perna et al. were able to compare drivers in vemurafenib-resistant and treatment-naïve tumors, and these authors identified novel mediators of vemurafenib-resistance including *Eras* [74]. *ERAS* is an activator of PI3K/AKT signaling, which the authors show fosters resistance to vemurafenib through inactivation of the pro-apoptotic protein BAD. Therefore, dual treatment with a PI3K inhibitor along with vemurafenib may be a promising treatment in the clinic if observed therapy-related toxicities can be overcome (NCT01512251). 

In another example, Kas et al. studied resistance to AZD4547, a selective FGFR inhibitor, by orthotopically implanting an *SB*-accelerated mammary tumor with *FGFR2* activation into syngeneic FVB mice and treating with AZD4547 [54]. FGFR is upstream of both the MAPK-ERK and PI3K-AKT pathways and is frequently hyperactivated in human cancers [131,132]. Clinical trials of several FGFR inhibitors have shown success in a subset of patients, but mechanisms of FGFR-inhibitor resistance are still being understood [133]. Treatment resistant versus naïve tumors were compared by RNA-sequencing and analysis of transposon insertion mutations. The authors identified a diverse spectrum of resistance mechanisms to FGFR inhibition. Reactivation of the MAPK-ERK pathway was the dominant form of resistance, suggesting that combining FGFR and MEK/ERK pathway inhibitors may be the most effective strategy for patients with FGFR activation. In addition, the authors found that *Abcg2*, a drug efflux pump, expression was upregulated in some AZD4547 treated tumors, while inactivation of *Rasa1* was found in other AZD4547 resistant tumors. These results provide guidance that future drug design of FGFR inhibitors should be specifically made to be poor substrates for drug efflux pumps. More pre-clinical models of *SB*-mediated accelerated tumor evolution could be used to predict drug resistance mechanisms in the clinic to give additional options to patients and facilitate more intelligent drug design (Figure 2B). Similarly, to the metastasis experiments described above, these experiments could be done using tumors derived from a small screen allografted into several recipient mice. Such studies would ideally be done in immunoproficient mice in the context of recurrent metastatic disease, to best approximate the clinical situation for patients. Drivers in therapy-resistant tumors would then be compared to untreated tumor drivers to find pathways involved in drug resistance.

In addition, a similar in vivo screen has been used to gain insight into resistance to the MET inhibitor Fortinib in a model of *SB*-accelerated medulloblastoma [98]. The *PB* system was used to identify mechanisms of resistance to an Mdm2 inhibitor in *PB*-accelerated tumors of various types passaged as allografts [134]. Although the following studies were not carried out in vivo, but rather in cell lines, it is worth noting that transposon mutagenesis has been successfully used to screen for cancer cell drug resistance in several reports [134,135]. Taken together, these studies suggest that cancer evolution in response to the selective pressures of therapy can be usefully explored using transposon mutagenesis.

## 7. Obesity and Tumor Development

Worldwide, the incidence of obesity has nearly tripled since 1975 [136]. Of particular concern, the prevalence of overweight and obese children ages 5-19 has risen from just 4% in 1975 to over 18% in 2016 [136]. The fundamental cause for this increase in obese and overweight people is an increase in energy-rich food intake and a reduction in activity. Excess adipose tissue predisposes individuals to develop type 2 diabetes mellitus, cardiovascular disease, and several types of cancer [137,138,139]. Obesity has been associated with increased cancer risk in colorectal, kidney, pancreatic, gallbladder, thyroid, breast, ovarian, esophageal, liver, and endometrial cancer [140,141,142]. Increased BMI (body mass index) has been associated with reduced cancer survival and increased recurrence after radio- or chemo-therapy [143,144,145].

However, despite strong clinical, preclinical, and epidemiological evidence linking obesity to increased cancer risk [138,139,146,147], the mechanisms behind this are still not completely understood. Local dysregulation in adipose tissues of obese individuals results in systemic metabolic changes including insulin resistance, chronic inflammation, and hyperglycemia [148,149]. Dysregulated paracrine signaling from adipocytes shapes a permissive microenvironment to tumor development and progression through secretion of signaling molecules (including proinflammatory cytokines, proangiogenic factors, and adipokines) and by acting as an energy reservoir [139,150,151,152]. For example, chronic inflammation brought on by obesity results in increased expression of signal transducer and activator of transcription 3 (STAT3) and nuclear factor-κB (NF-κB), which increase cellular proliferation and pro-survival gene expression [153,154,155]. Adipose tissue also hosts many immune cells which are significantly altered in the context of obesity [156,157]. 

Given the complex differences in the biology surrounding a cancer developing in the context of obesity, it is likely that the genetic drivers differ in these cancers. Transposon mutagenesis offers an opportunity to reflect these changes in how we model cancer in the mouse. For example, Tschida et al. used *SB* insertion mutagenesis to model hepatocellular carcinoma in the context of steatosis or accumulation of fat in the liver [27]. By comparing steatosis-associated drivers to drivers found in another screen with normal diet [65], the authors were able to identify steatosis-specific drivers. Many published screens should be repeated with the addition of diet-induced obesity in the mice and compared to available normal diet studies to identify targets specific to obesity (Figure 2C). While metastatic driver and therapy resistance studies can be done faithfully with orthotopically implanted tumors, we recommend diet-induced obesity screens be done with the full transposon mutagenesis process. While this is a larger undertaking, it will identify drivers of initiation and early progression in tumor formation, rather than just later drivers of progression and metastasis. These obesity-specific therapies are necessary to address the clearly unmet and increasing need for patients.

## 8. Conclusions and Future Directions

Transposon insertion mutagenesis is a powerful tool to facilitate accelerated evolution and further our understanding of the many dimensions of cancer development, progression, and response to therapy. In this review, we have covered some of the contributions made to the field of cancer biology. These have included models of many types of human cancers, providing insight into the genetic drivers of these cancers as well as powerful pre-clinical model systems. In these studies, the effects of increasing differentiation/linage commitment on cancer development have been studied, as have mechanisms of therapy resistance and metastasis. It was determined that the differentiation status of the tumor cell of origin affects the number of mutations required for tumor formation and the end-tumor expression patterns in surprising ways. Combination therapies have been proposed based on *SB* screens done in the context of a targeted therapy, such as dual treatment with a PI3K inhibitor and vemurafenib in melanoma and FGFR and MEK/ERK pathway inhibitors for breast cancer. In both osteosarcoma and medulloblastoma, drivers found in metastatic clones varied from those in other metastases as well as the primary tumor, indicating that targeted therapies based on the genetics of the primary tumor or even a single metastatic clone are unlikely to eliminate all metastases present.

In the future, we predict transposon mutagenesis will be used to mirror changes in our population by incorporating changes in nutrition in mouse models. This will allow more precise and appropriate therapy to be delivered to patients suffering from obesity in addition to cancer. In addition, transposon mutagenesis studies may help to suggest changes in cancer therapy by identifying resistance mechanisms to targeted therapies as described above and novel ideas for better drug design, including making drugs poor substrates for specific efflux pumps. We predict that transposon screens will be used to identify metastasis-specific drivers in additional tumor types providing additional treatment options for patients with high-risk disease. Lastly, we propose future screens to study the effects of aging on tumor development. For example, it would be interesting to compare screens with mutagenesis initiated in younger versus older mice (>1-year-old) through the use of tamoxifen-induced Cre. These studies may more accurately reflect the development of some tumor types that mainly occur in adult tissues but are poorly modeled by transposon-mutagenesis timed during embryogenesis or early development.

## Figures and Tables

**Table 1:** Systems for Cancer Functional Genomics.

Mutagenesis System	Advantages	Disadvantages
CRISPR/Cas9	Genome wide Useful in loss and gain of function studies Bias can be eliminated by careful guide RNA library design	Difficult to employ in primary cells, useful in only established cell lines Difficult to employ in vivo Difficult to select for phenotypes requiring multiple cooperating genetic alterations
Transposon	Genome wide Useful in loss and gain of function studies Allows screens to be done in cell lines or primary cells in vivo Useful for selection of traits requiring multiple cooperating mutations Non-coding or regulatory regions of the genome can be identified	Bias for or against parts of the genome due to local hopping and insertion site preference Some genes are unlikely to be activated by transposon insertion if first ATG is in exon 1 Some genes are unlikely to be inactivated due to their small size (e.g., microRNAs) Does not induce the full spectrum of mutations found in human cancers (e.g., point mutations and translocations) Transposon mutagenesis can create mutations not tagged by the transposon due to re-mobilization
Retroviruses	Many have been isolated with various tissue tropisms Can activate endogenous promoters by enhancement mechanisms Do not require generation of new transgenic lines of mice	Tend to not induce loss of function mutations, relatively few tumor suppressor genes identified in screens Systems generally found and not created, meaning there are no retroviruses useful for modeling many important types of cancer Tissue tropisms limit usefulness and types of cancer that can be modeled Generally, cells must be dividing for infection Many retroviruses have severe strain-specific effects and limitations

**Table 2:** *Published Sleeping Beauty* and *piggyBac* Cancer Screens in Mice.

Tumor Type	Transposase	Transposon	Cre	Sensitizing Mutations	Refs
*Sarcomas*					
Fibrosarcoma	*CGS-SB10*	*T2/Onc*	-	*p19arf*	[20]
Osteosarcoma	*R26-lsl-SB11*	*T2/Onc*	*Osx-Cre*	*Trp53*	[48]
Peripheral nerve sheath tumor	*R26-lsl-SB11*	*T2/Onc, T2/Onc15*	*Cnp-Cre, Dhh-Cre*	*Trp53, EGFR* *Nf1*	[29,49]
Histiocytic sarcoma	*R26-lsl-SB11*	*T2/Onc, T2/Onc2*	*Lyz2-Cre*	-	[50]
*Carcinomas*					
Skin	*K5-SB11*	*T2/Onc2*	-	*Hras*	[25]
Mammary	*K5-SB11, R26-lsl-SB11*	*T2/Onc2, T2/Onc3*	*K5-Cre, Wap-Cre*	*Trp53, β-catenin, Cdh1, FGFR, Pten*	[51,52,53,54,55]
Pancreatic	*R26-lsl-SB11, R26-lsl-SB13, R26-lsl-PB*	*T2/Onc, T2/Onc2, T2Onc3, ATP1*	*Pdx1-Cre*	*Kras*	[24,56,57]
Gastric adenoma	*R26-lsl-SB11*	*T2/Onc3*	*β-actin-Cre*	*Smad4*	[58]
Intestinal tract	*R26-lsl-SB11*	*T2/Onc, T2/Onc2*	*Vil-CreERT2, Vil-Cre, Ah-Cre*	*Apc, Kras, Smad4, Trp53, Tgfbr2*	[59,60,61,62,63]
Liver	*R26-lsl-SB11*	*T2/Onc, T2/Onc2, T2/Onc3*	*Alb-Cre*	*HBsAg, Trp53, Myc, Steatosis, Pten, Sav1, Met*	[27,64,65,66,67,68,69]
Lung	*R26-lsl-SB11*	*T2/Onc*	*Spc-Cre*	*Trp53, p19arf, Pten*	[70]
Prostate	*CGS-SB10, R26-SB11, R26-lsl-SB11*	*T2/Onc, T2/Onc3*	*PB-Cre*	*p19arf, Pten*	[71,72]
Thyroid	*R26-lsl-SB11*	*T2/Onc2*	*Tpo-Cre*	*Hras*	[73]
*Melanoma*	*R26-lsl-SB11, R26-lsl-SB13, Act-PBase*	*T2/Onc, T2/Onc2, T2/Onc3* *Luc-PB[mut]7*	*Tyr-Cre-ERT2*	*Braf*	[74,75,76,77]
*Hematopoietic*					
T cell leukemia	*R26-lsl-SB11, R26-SB11*	*T2/Onc2*	*Vav-iCre, Lck-Cre, CD4-Cre*	-	[21,78]
T cell lymphoma	*R26-SB11, R26-lsl-PB*	*T2/Onc, ATP2*	*CD4-Cre*	*Trp53, ITK-SYK, Pdc1*	[79,80]
B cell leukemia	*R26-lsl-SB11, Etv6-RUNX1-HSB5*	*T2/Onc*	*Cd79a-Cre*	*Stat5b, Etv6-RUNX1 fusion*	[81,82]
B cell lymphoma	*R26-lsl-SB11, Etv6-RUNX1-HSB5, R26-PB*	*T2/Onc, T2/Onc2, T2/Onc15, ITP1, ITP2*	*Aid-Cre, CD19-Cre, Cnp-Cre*	*Eμ-TCL1, Pax5, Etv6-RUNX1 fusion, Trp53, Pten, Blm*	[23,83,84,85,86]
Acute myeloid leukemia	*R26-lsl-SB11*	*T2/Onc2, GrOnc*	*β-actin-Cre, Vav-Cre, Mx1-Cre*	*Trp53, Jak2, Npm1c, BCR-ABL*	[87,88,89,90]
Mixture of T cell and B cell lymphoma, myeloid leukemia	*R26-SB11*	*T2/Onc*	*-*	*Rassf1a, Cadm1*	[91,92]
Erythroleukemia	*R26-lsl-SB11*	*T2/Onc2*	*Mx1-Cre*	*Cyclin E*	[93]
Myeloid and lymphoid malignancies, thymus, spleen	*R26-lsl-SB11*	*T2/Onc2*	*Vec-Cre*	-	[94]
*Brain tumors*					
Medulloblastoma/CNS-ET *	*R26-lsl-SB11, R26-SB11, Math1-SB11*	*T2/Onc, T2/Onc2, T2/Onc3*	*β-actin-Cre, Nestin-Cre*	*Ptch1, Trp53, Pten*	[28,95,96,97,98,99]
Glioma	*R26-lsl-SB11, R26-SB11*	*T2/Onc, T2/Onc2, T2/Onc3, T2OncATG*	*Nestin-Cre*	*Trp53, p19Arf, Blm, Csf1*	[26,100,101]
*Multiple tumor types*					
Skin, brain, airway, liver, leukemia, lymphoma, intestine	*R26-SB11*	*T2/Onc3*	*-*	*Rag2*	[102]
Leukemia, medulloblastoma, glioma	*CGS-SB10, R26-SB11*	*T2/Onc*	*-*	*p19Arf*	[22]
Skin, liver, lung, brain, lymphoma, sarcoma, mammary, colon, etc.	*R26-SB11*	*T2/Onc3*	-	-	[23]
T cell and B cell leukemia, lymphoma, skin, sarcoma, intestinal tract, lung, liver, etc.	*R26-PB*	*ATP1, ATP2, ATP3*	-	-	[103]
Prostate, mammary and skin carcinomas	*R26-SB11*	*ITP2m*	*-*	*Pten, Blm*	[104]
Sarcoma, carcinoma, leukemia, resistance to MDM2 inhibition	*R26-PB*	*ATP2*	*-*	*p19Arf*	[105]
Liver, lung carcinoma, skin carcinoma, lymphoma	*R26-PB*	*ATP1*	-	-	[106]

* CNS-ET—Embryonal tumor of the central nervous system.

## References

[1] Mc C.B. (1950). The origin and behavior of mutable loci in maize. Proc. Natl. Acad. Sci. USA.

[2] Paatero A.O., Turakainen H., Happonen L.J., Olsson C., Palomaki T., Pajunen M.I., Meng X., Otonkoski T., Tuuri T., Berry C. (2008). Bacteriophage Mu integration in yeast and mammalian genomes. Nucleic Acids Res..

[3] Wisman E., Cardon G.H., Fransz P., Saedler H. (1998). The behaviour of the autonomous maize transposable element En/Spm in Arabidopsis thaliana allows efficient mutagenesis. Plant Mol. Biol..

[4] Enoki H., Izawa T., Kawahara M., Komatsu M., Koh S., Kyozuka J., Shimamoto K. (1999). Ac as a tool for the functional genomics of rice. Plant J..

[5] Meissner R., Chague V., Zhu Q., Emmanuel E., Elkind Y., Levy A.A. (2000). Technical advance: A high throughput system for transposon tagging and promoter trapping in tomato. Plant J..

[6] Kuromori T., Hirayama T., Kiyosue Y., Takabe H., Mizukado S., Sakurai T., Akiyama K., Kamiya A., Ito T., Shinozaki K. (2004). A collection of 11 800 single-copy Ds transposon insertion lines in Arabidopsis. Plant J..

[7] Greco R., Ouwerkerk P.B., Sallaud C., Kohli A., Colombo L., Puigdomenech P., Guiderdoni E., Christou P., Hoge J.H., Pereira A. (2001). Transposon insertional mutagenesis in rice. Plant Physiol..

[8] Ruaud A.F., Bessereau J.L. (2006). Activation of nicotinic receptors uncouples a developmental timer from the molting timer in C. elegans. Development.

[9] Ruaud A.F., Bessereau J.L. (2007). The P-type ATPase CATP-1 is a novel regulator of C. elegans developmental timing that acts independently of its predicted pump function. Development.

[10] Gally C., Eimer S., Richmond J.E., Bessereau J.L. (2004). A transmembrane protein required for acetylcholine receptor clustering in Caenorhabditis elegans. Nature.

[11] Yook K., Hodgkin J. (2007). Mos1 mutagenesis reveals a diversity of mechanisms affecting response of Caenorhabditis elegans to the bacterial pathogen Microbacterium nematophilum. Genetics.

[12] Hummel T., Klambt C. (2008). P-element mutagenesis. Methods Mol. Biol..

[13] Handler A.M., Harrell R.A. (1999). Germline transformation of Drosophila melanogaster with the piggyBac transposon vector. Insect Mol. Biol..

[14] Loukeris T.G., Arca B., Livadaras I., Dialektaki G., Savakis C. (1995). Introduction of the transposable element Minos into the germ line of Drosophila melanogaster. Proc. Natl. Acad. Sci. USA.

[15] Urasaki A., Mito T., Noji S., Ueda R., Kawakami K. (2008). Transposition of the vertebrate Tol2 transposable element in Drosophila melanogaster. Gene.

[16] Ding S., Wu X., Li G., Han M., Zhuang Y., Xu T. (2005). Efficient transposition of the piggyBac (PB) transposon in mammalian cells and mice. Cell.

[17] Drabek D., Zagoraiou L., deWit T., Langeveld A., Roumpaki C., Mamalaki C., Savakis C., Grosveld F. (2003). Transposition of the Drosophila hydei Minos transposon in the mouse germ line. Genomics.

[18] Kawakami K., Shima A., Kawakami N. (2000). Identification of a functional transposase of the Tol2 element, an Ac-like element from the Japanese medaka fish, and its transposition in the zebrafish germ lineage. Proc. Natl. Acad. Sci. USA.

[19] Ivics Z., Hackett P.B., Plasterk R.H., Izsvak Z. (1997). Molecular reconstruction of Sleeping Beauty, a Tc1-like transposon from fish, and its transposition in human cells. Cell.

[20] Collier L.S., Carlson C.M., Ravimohan S., Dupuy A.J., Largaespada D.A. (2005). Cancer gene discovery in solid tumours using transposon-based somatic mutagenesis in the mouse. Nature.

[21] Dupuy A.J., Akagi K., Largaespada D.A., Copeland N.G., Jenkins N.A. (2005). Mammalian mutagenesis using a highly mobile somatic Sleeping Beauty transposon system. Nature.

[22] Collier L.S., Adams D.J., Hackett C.S., Bendzick L.E., Akagi K., Davies M.N., Diers M.D., Rodriguez F.J., Bender A.M., Tieu C. (2009). Whole-body sleeping beauty mutagenesis can cause penetrant leukemia/lymphoma and rare high-grade glioma without associated embryonic lethality. Cancer Res..

[23] Dupuy A.J., Rogers L.M., Kim J., Nannapaneni K., Starr T.K., Liu P., Largaespada D.A., Scheetz T.E., Jenkins N.A., Copeland N.G. (2009). A modified sleeping beauty transposon system that can be used to model a wide variety of human cancers in mice. Cancer Res..

[24] Perez-Mancera P.A., Rust A.G., van der Weyden L., Kristiansen G., Li A., Sarver A.L., Silverstein K.A., Grutzmann R., Aust D., Rummele P. (2012). The deubiquitinase USP9X suppresses pancreatic ductal adenocarcinoma. Nature.

[25] Quintana R.M., Dupuy A.J., Bravo A., Casanova M.L., Alameda J.P., Page A., Sanchez-Viera M., Ramirez A., Navarro M. (2013). A transposon-based analysis of gene mutations related to skin cancer development. J. Investig. Dermatol..

[26] Vyazunova I., Maklakova V.I., Berman S., De I., Steffen M.D., Hong W., Lincoln H., Morrissy A.S., Taylor M.D., Akagi K. (2014). Sleeping Beauty mouse models identify candidate genes involved in gliomagenesis. PLoS ONE.

[27] Tschida B.R., Temiz N.A., Kuka T.P., Lee L.A., Riordan J.D., Tierrablanca C.A., Hullsiek R., Wagner S., Hudson W.A., Linden M.A. (2017). Sleeping Beauty Insertional Mutagenesis in Mice Identifies Drivers of Steatosis-Associated Hepatic Tumors. Cancer Res..

[28] Beckmann P.J., Larson J.D., Larsson A.T., Ostergaard J.P., Wagner S., Rahrmann E.P., Shamsan G.A., Otto G.M., Williams R.L., Wang J. (2019). Sleeping Beauty Insertional Mutagenesis Reveals Important Genetic Drivers of Central Nervous System Embryonal Tumors. Cancer Res..

[29] Rahrmann E.P., Watson A.L., Keng V.W., Choi K., Moriarity B.S., Beckmann D.A., Wolf N.K., Sarver A., Collins M.H., Moertel C.L. (2013). Forward genetic screen for malignant peripheral nerve sheath tumor formation identifies new genes and pathways driving tumorigenesis. Nat. Genet..

[30] Kitada K., Ishishita S., Tosaka K., Takahashi R., Ueda M., Keng V.W., Horie K., Takeda J. (2007). Transposon-tagged mutagenesis in the rat. Nat. Methods.

[31] Nasevicius A., Ekker S.C. (2000). Effective targeted gene ‘knockdown’ in zebrafish. Nat. Genet..

[32] Sinzelle L., Vallin J., Coen L., Chesneau A., Du Pasquier D., Pollet N., Demeneix B., Mazabraud A. (2006). Generation of trangenic Xenopus laevis using the Sleeping Beauty transposon system. Transgenic Res..

[33] Izsvak Z., Ivics Z., Plasterk R.H. (2000). Sleeping Beauty, a wide host-range transposon vector for genetic transformation in vertebrates. J. Mol. Biol..

[34] Geurts A.M., Yang Y., Clark K.J., Liu G., Cui Z., Dupuy A.J., Bell J.B., Largaespada D.A., Hackett P.B. (2003). Gene transfer into genomes of human cells by the sleeping beauty transposon system. Mol. Ther..

[35] Karsi A., Moav B., Hackett P., Liu Z. (2001). Effects of insert size on transposition efficiency of the sleeping beauty transposon in mouse cells. Mar. Biotechnol..

[36] Rostovskaya M., Fu J., Obst M., Baer I., Weidlich S., Wang H., Smith A.J., Anastassiadis K., Stewart A.F. (2012). Transposon-mediated BAC transgenesis in human ES cells. Nucleic Acids Res..

[37] Zayed H., Izsvak Z., Walisko O., Ivics Z. (2004). Development of hyperactive sleeping beauty transposon vectors by mutational analysis. Mol. Ther..

[38] Balciunas D., Wangensteen K.J., Wilber A., Bell J., Geurts A., Sivasubbu S., Wang X., Hackett P.B., Largaespada D.A., McIvor R.S. (2006). Harnessing a high cargo-capacity transposon for genetic applications in vertebrates. PLoS Genet..

[39] Wang W., Lin C., Lu D., Ning Z., Cox T., Melvin D., Wang X., Bradley A., Liu P. (2008). Chromosomal transposition of PiggyBac in mouse embryonic stem cells. Proc. Natl. Acad. Sci. USA.

[40] Yant S.R., Wu X., Huang Y., Garrison B., Burgess S.M., Kay M.A. (2005). High-resolution genome-wide mapping of transposon integration in mammals. Mol. Cell Biol..

[41] Liu G., Geurts A.M., Yae K., Srinivasan A.R., Fahrenkrug S.C., Largaespada D.A., Takeda J., Horie K., Olson W.K., Hackett P.B. (2005). Target-site preferences of Sleeping Beauty transposons. J. Mol. Biol..

[42] Fischer S.E., Wienholds E., Plasterk R.H. (2001). Regulated transposition of a fish transposon in the mouse germ line. Proc. Natl. Acad. Sci. USA.

[43] Weber J., Ollinger R., Friedrich M., Ehmer U., Barenboim M., Steiger K., Heid I., Mueller S., Maresch R., Engleitner T. (2015). CRISPR/Cas9 somatic multiplex-mutagenesis for high-throughput functional cancer genomics in mice. Proc. Natl. Acad. Sci. USA.

[44] Xu C., Qi X., Du X., Zou H., Gao F., Feng T., Lu H., Li S., An X., Zhang L. (2017). piggyBac mediates efficient in vivo CRISPR library screening for tumorigenesis in mice. Proc. Natl. Acad. Sci. USA.

[45] Kool J., Berns A. (2009). High-throughput insertional mutagenesis screens in mice to identify oncogenic networks. Nat. Rev. Cancer.

[46] Theodorou V., Kimm M.A., Boer M., Wessels L., Theelen W., Jonkers J., Hilkens J. (2007). MMTV insertional mutagenesis identifies genes, gene families and pathways involved in mammary cancer. Nat. Genet..

[47] Laboratory T.J. Characterized Cre Lines. https://www.jax.org/research-and-faculty/resources/cre-repository/characterized-cre-lines-jax-cre-resource#.

[48] Moriarity B.S., Otto G.M., Rahrmann E.P., Rathe S.K., Wolf N.K., Weg M.T., Manlove L.A., LaRue R.S., Temiz N.A., Molyneux S.D. (2015). A Sleeping Beauty forward genetic screen identifies new genes and pathways driving osteosarcoma development and metastasis. Nat. Genet..

[49] Wu J., Keng V.W., Patmore D.M., Kendall J.J., Patel A.V., Jousma E., Jessen W.J., Choi K., Tschida B.R., Silverstein K.A. (2016). Insertional Mutagenesis Identifies a STAT3/Arid1b/beta-catenin Pathway Driving Neurofibroma Initiation. Cell Rep..

[50] Been R.A., Linden M.A., Hager C.J., DeCoursin K.J., Abrahante J.E., Landman S.R., Steinbach M., Sarver A.L., Largaespada D.A., Starr T.K. (2014). Genetic signature of histiocytic sarcoma revealed by a sleeping beauty transposon genetic screen in mice. PLoS ONE.

[51] Suarez-Cabrera C., Quintana R.M., Bravo A., Casanova M.L., Page A., Alameda J.P., Paramio J.M., Maroto A., Salamanca J., Dupuy A.J. (2017). A Transposon-based Analysis Reveals RASA1 Is Involved in Triple-Negative Breast Cancer. Cancer Res..

[52] Chen L., Jenjaroenpun P., Pillai A.M., Ivshina A.V., Ow G.S., Efthimios M., Zhiqun T., Tan T.Z., Lee S.C., Rogers K. (2017). Transposon insertional mutagenesis in mice identifies human breast cancer susceptibility genes and signatures for stratification. Proc. Natl. Acad. Sci. USA.

[53] Kas S.M., de Ruiter J.R., Schipper K., Annunziato S., Schut E., Klarenbeek S., Drenth A.P., van der Burg E., Klijn C., Ten Hoeve J.J. (2017). Insertional mutagenesis identifies drivers of a novel oncogenic pathway in invasive lobular breast carcinoma. Nat. Genet..

[54] Kas S.M., de Ruiter J.R., Schipper K., Schut E., Bombardelli L., Wientjens E., Drenth A.P., de Korte-Grimmerink R., Mahakena S., Phillips C. (2018). Transcriptomics and Transposon Mutagenesis Identify Multiple Mechanisms of Resistance to the FGFR Inhibitor AZD4547. Cancer Res..

[55] Rangel R., Lee S.C., Hon-Kim Ban K., Guzman-Rojas L., Mann M.B., Newberg J.Y., Kodama T., McNoe L.A., Selvanesan L., Ward J.M. (2016). Transposon mutagenesis identifies genes that cooperate with mutant Pten in breast cancer progression. Proc. Natl. Acad. Sci. USA.

[56] Mann K.M., Ward J.M., Yew C.C., Kovochich A., Dawson D.W., Black M.A., Brett B.T., Sheetz T.E., Dupuy A.J., Chang D.K. (2012). Sleeping Beauty mutagenesis reveals cooperating mutations and pathways in pancreatic adenocarcinoma. Proc. Natl. Acad. Sci. USA.

[57] Rad R., Rad L., Wang W., Strong A., Ponstingl H., Bronner I.F., Mayho M., Steiger K., Weber J., Hieber M. (2015). A conditional piggyBac transposition system for genetic screening in mice identifies oncogenic networks in pancreatic cancer. Nat. Genet..

[58] Takeda H., Rust A.G., Ward J.M., Yew C.C., Jenkins N.A., Copeland N.G. (2016). Sleeping Beauty transposon mutagenesis identifies genes that cooperate with mutant Smad4 in gastric cancer development. Proc. Natl. Acad. Sci. USA.

[59] Takeda H., Wei Z., Koso H., Rust A.G., Yew C.C., Mann M.B., Ward J.M., Adams D.J., Copeland N.G., Jenkins N.A. (2015). Transposon mutagenesis identifies genes and evolutionary forces driving gastrointestinal tract tumor progression. Nat. Genet..

[60] Starr T.K., Allaei R., Silverstein K.A., Staggs R.A., Sarver A.L., Bergemann T.L., Gupta M., O’Sullivan M.G., Matise I., Dupuy A.J. (2009). A transposon-based genetic screen in mice identifies genes altered in colorectal cancer. Science.

[61] Starr T.K., Scott P.M., Marsh B.M., Zhao L., Than B.L., O’Sullivan M.G., Sarver A.L., Dupuy A.J., Largaespada D.A., Cormier R.T. (2011). A Sleeping Beauty transposon-mediated screen identifies murine susceptibility genes for adenomatous polyposis coli (Apc)-dependent intestinal tumorigenesis. Proc. Natl. Acad. Sci. USA.

[62] March H.N., Rust A.G., Wright N.A., ten Hoeve J., de Ridder J., Eldridge M., van der Weyden L., Berns A., Gadiot J., Uren A. (2011). Insertional mutagenesis identifies multiple networks of cooperating genes driving intestinal tumorigenesis. Nat. Genet..

[63] Morris S.M., Davison J., Carter K.T., O’Leary R.M., Trobridge P., Knoblaugh S.E., Myeroff L.L., Markowitz S.D., Brett B.T., Scheetz T.E. (2017). Transposon mutagenesis identifies candidate genes that cooperate with loss of transforming growth factor-beta signaling in mouse intestinal neoplasms. Int. J. Cancer.

[64] Bard-Chapeau E.A., Nguyen A.T., Rust A.G., Sayadi A., Lee P., Chua B.Q., New L.S., de Jong J., Ward J.M., Chin C.K. (2014). Transposon mutagenesis identifies genes driving hepatocellular carcinoma in a chronic hepatitis B mouse model. Nat. Genet..

[65] Keng V.W., Villanueva A., Chiang D.Y., Dupuy A.J., Ryan B.J., Matise I., Silverstein K.A., Sarver A., Starr T.K., Akagi K. (2009). A conditional transposon-based insertional mutagenesis screen for genes associated with mouse hepatocellular carcinoma. Nat. Biotechnol..

[66] Keng V.W., Sia D., Sarver A.L., Tschida B.R., Fan D., Alsinet C., Sole M., Lee W.L., Kuka T.P., Moriarity B.S. (2013). Sex bias occurrence of hepatocellular carcinoma in Poly7 molecular subclass is associated with EGFR. Hepatology.

[67] O’Donnell K.A., Keng V.W., York B., Reineke E.L., Seo D., Fan D., Silverstein K.A., Schrum C.T., Xie W.R., Mularoni L. (2012). A Sleeping Beauty mutagenesis screen reveals a tumor suppressor role for Ncoa2/Src-2 in liver cancer. Proc. Natl. Acad. Sci. USA.

[68] Kodama T., Yi J., Newberg J.Y., Tien J.C., Wu H., Finegold M.J., Kodama M., Wei Z., Tamura T., Takehara T. (2018). Molecular profiling of nonalcoholic fatty liver disease-associated hepatocellular carcinoma using SB transposon mutagenesis. Proc. Natl. Acad. Sci. USA.

[69] Fan Y., Bazai S.K., Daian F., Arechederra M., Richelme S., Temiz N.A., Yim A., Habermann B.H., Dono R., Largaespada D.A. (2019). Evaluating the landscape of gene cooperativity with receptor tyrosine kinases in liver tumorigenesis using transposon-mediated mutagenesis. J. Hepatol..

[70] Dorr C., Janik C., Weg M., Been R.A., Bader J., Kang R., Ng B., Foran L., Landman S.R., O’Sullivan M.G. (2015). Transposon Mutagenesis Screen Identifies Potential Lung Cancer Drivers and CUL3 as a Tumor Suppressor. Mol. Cancer Res..

[71] Rahrmann E.P., Collier L.S., Knutson T.P., Doyal M.E., Kuslak S.L., Green L.E., Malinowski R.L., Roethe L., Akagi K., Waknitz M. (2009). Identification of PDE4D as a proliferation promoting factor in prostate cancer using a Sleeping Beauty transposon-based somatic mutagenesis screen. Cancer Res..

[72] Ahmad I., Mui E., Galbraith L., Patel R., Tan E.H., Salji M., Rust A.G., Repiscak P., Hedley A., Markert E. (2016). Sleeping Beauty screen reveals Pparg activation in metastatic prostate cancer. Proc. Natl. Acad. Sci. USA.

[73] Montero-Conde C., Leandro-Garcia L.J., Chen X., Oler G., Ruiz-Llorente S., Ryder M., Landa I., Sanchez-Vega F., La K., Ghossein R.A. (2017). Transposon mutagenesis identifies chromatin modifiers cooperating with Ras in thyroid tumorigenesis and detects ATXN7 as a cancer gene. Proc. Natl. Acad. Sci. USA.

[74] Perna D., Karreth F.A., Rust A.G., Perez-Mancera P.A., Rashid M., Iorio F., Alifrangis C., Arends M.J., Bosenberg M.W., Bollag G. (2015). BRAF inhibitor resistance mediated by the AKT pathway in an oncogenic BRAF mouse melanoma model. Proc. Natl. Acad. Sci. USA.

[75] Mann M.B., Black M.A., Jones D.J., Ward J.M., Yew C.C., Newberg J.Y., Dupuy A.J., Rust A.G., Bosenberg M.W., McMahon M. (2015). Transposon mutagenesis identifies genetic drivers of Braf melanoma. Nat. Genet..

[76] Karreth F.A., Tay Y., Perna D., Ala U., Tan S.M., Rust A.G., DeNicola G., Webster K.A., Weiss D., Perez-Mancera P.A. (2011). In vivo identification of tumor- suppressive PTEN ceRNAs in an oncogenic BRAF-induced mouse model of melanoma. Cell.

[77] Ni T.K., Landrette S.F., Bjornson R.D., Bosenberg M.W., Xu T. (2013). Low-copy piggyBac transposon mutagenesis in mice identifies genes driving melanoma. Proc. Natl. Acad. Sci. USA.

[78] Berquam-Vrieze K.E., Nannapaneni K., Brett B.T., Holmfeldt L., Ma J., Zagorodna O., Jenkins N.A., Copeland N.G., Meyerholz D.K., Knudson C.M. (2011). Cell of origin strongly influences genetic selection in a mouse model of T-ALL. Blood.

[79] van der Weyden L., Rust A.G., McIntyre R.E., Robles-Espinoza C.D., del Castillo Velasco-Herrera M., Strogantsev R., Ferguson-Smith A.C., McCarthy S., Keane T.M., Arends M.J. (2013). Jdp2 downregulates Trp53 transcription to promote leukaemogenesis in the context of Trp53 heterozygosity. Oncogene.

[80] Wartewig T., Kurgyis Z., Keppler S., Pechloff K., Hameister E., Ollinger R., Maresch R., Buch T., Steiger K., Winter C. (2017). PD-1 is a haploinsufficient suppressor of T cell lymphomagenesis. Nature.

[81] Heltemes-Harris L.M., Larson J.D., Starr T.K., Hubbard G.K., Sarver A.L., Largaespada D.A., Farrar M.A. (2015). Sleeping Beauty transposon screen identifies signaling modules that cooperate with STAT5 activation to induce B-cell acute lymphoblastic leukemia. Oncogene.

[82] van der Weyden L., Giotopoulos G., Rust A.G., Matheson L.S., van Delft F.W., Kong J., Corcoran A.E., Greaves M.F., Mullighan C.G., Huntly B.J. (2011). Modeling the evolution of ETV6-RUNX1-induced B-cell precursor acute lymphoblastic leukemia in mice. Blood.

[83] Zanesi N., Balatti V., Riordan J., Burch A., Rizzotto L., Palamarchuk A., Cascione L., Lagana A., Dupuy A.J., Croce C.M. (2013). A Sleeping Beauty screen reveals NF-kB activation in CLL mouse model. Blood.

[84] van der Weyden L., Giotopoulos G., Wong K., Rust A.G., Robles-Espinoza C.D., Osaki H., Huntly B.J., Adams D.J. (2015). Somatic drivers of B-ALL in a model of ETV6-RUNX1; Pax5(+/-) leukemia. BMC Cancer.

[85] Rahrmann E.P., Wolf N.K., Otto G.M., Heltemes-Harris L., Ramsey L.B., Shu J., LaRue R.S., Linden M.A., Rathe S.K., Starr T.K. (2019). Sleeping Beauty Screen Identifies RREB1 and Other Genetic Drivers in Human B-cell Lymphoma. Mol. Cancer Res..

[86] Weber J., de la Rosa J., Grove C.S., Schick M., Rad L., Baranov O., Strong A., Pfaus A., Friedrich M.J., Engleitner T. (2019). PiggyBac transposon tools for recessive screening identify B-cell lymphoma drivers in mice. Nat. Commun..

[87] Mann K.M., Newberg J.Y., Black M.A., Jones D.J., Amaya-Manzanares F., Guzman-Rojas L., Kodama T., Ward J.M., Rust A.G., van der Weyden L. (2016). Analyzing tumor heterogeneity and driver genes in single myeloid leukemia cells with SBCapSeq. Nat. Biotechnol..

[88] Tang J.Z., Carmichael C.L., Shi W., Metcalf D., Ng A.P., Hyland C.D., Jenkins N.A., Copeland N.G., Howell V.M., Zhao Z.J. (2013). Transposon mutagenesis reveals cooperation of ETS family transcription factors with signaling pathways in erythro-megakaryocytic leukemia. Proc. Natl. Acad. Sci. USA.

[89] Vassiliou G.S., Cooper J.L., Rad R., Li J., Rice S., Uren A., Rad L., Ellis P., Andrews R., Banerjee R. (2011). Mutant nucleophosmin and cooperating pathways drive leukemia initiation and progression in mice. Nat. Genet..

[90] Giotopoulos G., van der Weyden L., Osaki H., Rust A.G., Gallipoli P., Meduri E., Horton S.J., Chan W.I., Foster D., Prinjha R.K. (2015). A novel mouse model identifies cooperating mutations and therapeutic targets critical for chronic myeloid leukemia progression. J. Exp. Med..

[91] van der Weyden L., Papaspyropoulos A., Poulogiannis G., Rust A.G., Rashid M., Adams D.J., Arends M.J., O’Neill E. (2012). Loss of RASSF1A synergizes with deregulated RUNX2 signaling in tumorigenesis. Cancer Res..

[92] van der Weyden L., Arends M.J., Rust A.G., Poulogiannis G., McIntyre R.E., Adams D.J. (2012). Increased tumorigenesis associated with loss of the tumor suppressor gene Cadm1. Mol. Cancer.

[93] Loeb K.R., Hughes B.T., Fissel B.M., Osteen N.J., Knoblaugh S.E., Grim J.E., Drury L.J., Sarver A., Dupuy A.J., Clurman B.E. (2019). Insertional mutagenesis using the Sleeping Beauty transposon system identifies drivers of erythroleukemia in mice. Sci. Rep..

[94] Ziyad S., Riordan J.D., Cavanaugh A.M., Su T., Hernandez G.E., Hilfenhaus G., Morselli M., Huynh K., Wang K., Chen J.N. (2018). A Forward Genetic Screen Targeting the Endothelium Reveals a Regulatory Role for the Lipid Kinase Pi4ka in Myelo- and Erythropoiesis. Cell Rep..

[95] Genovesi L.A., Ng C.G., Davis M.J., Remke M., Taylor M.D., Adams D.J., Rust A.G., Ward J.M., Ban K.H., Jenkins N.A. (2013). Sleeping Beauty mutagenesis in a mouse medulloblastoma model defines networks that discriminate between human molecular subgroups. Proc. Natl. Acad. Sci. USA.

[96] Koso H., Tsuhako A., Lyons E., Ward J.M., Rust A.G., Adams D.J., Jenkins N.A., Copeland N.G., Watanabe S. (2014). Identification of FoxR2 as an oncogene in medulloblastoma. Cancer Res..

[97] Lastowska M., Al-Afghani H., Al-Balool H.H., Sheth H., Mercer E., Coxhead J.M., Redfern C.P., Peters H., Burt A.D., Santibanez-Koref M. (2013). Identification of a neuronal transcription factor network involved in medulloblastoma development. Acta Neuropathol. Commun..

[98] Bertrand K.C., Faria C.C., Skowron P., Luck A., Garzia L., Wu X., Agnihotri S., Smith C.A., Taylor M.D., Mack S.C. (2018). A functional genomics approach to identify pathways of drug resistance in medulloblastoma. Acta Neuropathol. Commun..

[99] Wu X., Northcott P.A., Dubuc A., Dupuy A.J., Shih D.J., Witt H., Croul S., Bouffet E., Fults D.W., Eberhart C.G. (2012). Clonal selection drives genetic divergence of metastatic medulloblastoma. Nature.

[100] Koso H., Takeda H., Yew C.C., Ward J.M., Nariai N., Ueno K., Nagasaki M., Watanabe S., Rust A.G., Adams D.J. (2012). Transposon mutagenesis identifies genes that transform neural stem cells into glioma-initiating cells. Proc. Natl. Acad. Sci. USA.

[101] Bender A.M., Collier L.S., Rodriguez F.J., Tieu C., Larson J.D., Halder C., Mahlum E., Kollmeyer T.M., Akagi K., Sarkar G. (2010). Sleeping beauty-mediated somatic mutagenesis implicates CSF1 in the formation of high-grade astrocytomas. Cancer Res..

[102] Rogers L.M., Olivier A.K., Meyerholz D.K., Dupuy A.J. (2013). Adaptive immunity does not strongly suppress spontaneous tumors in a Sleeping Beauty model of cancer. J. Immunol..

[103] Rad R., Rad L., Wang W., Cadinanos J., Vassiliou G., Rice S., Campos L.S., Yusa K., Banerjee R., Li M.A. (2010). PiggyBac transposon mutagenesis: A tool for cancer gene discovery in mice. Science.

[104] de la Rosa J., Weber J., Friedrich M.J., Li Y., Rad L., Ponstingl H., Liang Q., de Quiros S.B., Noorani I., Metzakopian E. (2017). A single-copy Sleeping Beauty transposon mutagenesis screen identifies new PTEN-cooperating tumor suppressor genes. Nat. Genet..

[105] Chapeau E.A., Gembarska A., Durand E.Y., Mandon E., Estadieu C., Romanet V., Wiesmann M., Tiedt R., Lehar J., de Weck A. (2017). Resistance mechanisms to TP53-MDM2 inhibition identified by in vivo piggyBac transposon mutagenesis screen in an Arf-/- mouse model. Proc. Natl. Acad. Sci. USA.

[106] Friedel R.H., Friedel C.C., Bonfert T., Shi R., Rad R., Soriano P. (2013). Clonal expansion analysis of transposon insertions by high-throughput sequencing identifies candidate cancer genes in a PiggyBac mutagenesis screen. PLoS ONE.

[107] Barker N., Ridgway R.A., van Es J.H., van de Wetering M., Begthel H., van den Born M., Danenberg E., Clarke A.R., Sansom O.J., Clevers H. (2009). Crypt stem cells as the cells-of-origin of intestinal cancer. Nature.

[108] Yang Z.J., Ellis T., Markant S.L., Read T.A., Kessler J.D., Bourboulas M., Schuller U., Machold R., Fishell G., Rowitch D.H. (2008). Medulloblastoma can be initiated by deletion of Patched in lineage-restricted progenitors or stem cells. Cancer Cell.

[109] Leiserson M.D., Vandin F., Wu H.T., Dobson J.R., Eldridge J.V., Thomas J.L., Papoutsaki A., Kim Y., Niu B., McLellan M. (2015). Pan-cancer network analysis identifies combinations of rare somatic mutations across pathways and protein complexes. Nat. Genet..

[110] Sturm D., Orr B.A., Toprak U.H., Hovestadt V., Jones D.T.W., Capper D., Sill M., Buchhalter I., Northcott P.A., Leis I. (2016). New Brain Tumor Entities Emerge from Molecular Classification of CNS-PNETs. Cell.

[111] Song H., He W., Huang X., Zhang H., Huang T. (2016). High expression of FOXR2 in breast cancer correlates with poor prognosis. Tumour Biol..

[112] Wang X., He B., Gao Y., Li Y. (2016). FOXR2 contributes to cell proliferation and malignancy in human hepatocellular carcinoma. Tumour Biol..

[113] Xu W., Chang J., Liu G., Du X., Li X. (2017). Knockdown of FOXR2 suppresses the tumorigenesis, growth and metastasis of prostate cancer. Biomed. Pharmacother..

[114] Abbott K.L., Nyre E.T., Abrahante J., Ho Y.Y., Isaksson Vogel R., Starr T.K. (2015). The Candidate Cancer Gene Database: A database of cancer driver genes from forward genetic screens in mice. Nucleic Acids Res..

[115] Sun W.L., Wang L., Luo J., Zhu H.W., Cai Z.W. (2018). Ambra1 modulates the sensitivity of breast cancer cells to epirubicin by regulating autophagy via ATG12. Cancer Sci..

[116] Li X., Zhang L., Yu L., Wei W., Lin X., Hou X., Tian Y. (2016). shRNA-mediated AMBRA1 knockdown reduces the cisplatin-induced autophagy and sensitizes ovarian cancer cells to cisplatin. J. Toxicol. Sci..

[117] Sun W.L. (2016). Ambra1 in autophagy and apoptosis: Implications for cell survival and chemotherapy resistance. Oncol. Lett..

[118] Bondy-Chorney E., Baldwin R.M., Didillon A., Chabot B., Jasmin B.J., Cote J. (2017). RNA binding protein RALY promotes Protein Arginine Methyltransferase 1 alternatively spliced isoform v2 relative expression and metastatic potential in breast cancer cells. Int. J. Biochem. Cell Biol..

[119] Zhu Z., Zhang Y., Huang C., Tang Y., Sun C., Ju W., He X. (2018). Overexpression of RALY promotes migration and predicts poor prognosis in hepatocellular carcinoma. Cancer Manag. Res..

[120] Tsofack S.P., Garand C., Sereduk C., Chow D., Aziz M., Guay D., Yin H.H., Lebel M. (2011). NONO and RALY proteins are required for YB-1 oxaliplatin induced resistance in colon adenocarcinoma cell lines. Mol. Cancer.

[121] Russell J.P., Powell D.J., Cunnane M., Greco A., Portella G., Santoro M., Fusco A., Rothstein J.L. (2000). The TRK-T1 fusion protein induces neoplastic transformation of thyroid epithelium. Oncogene.

[122] Tognon C., Knezevich S.R., Huntsman D., Roskelley C.D., Melnyk N., Mathers J.A., Becker L., Carneiro F., MacPherson N., Horsman D. (2002). Expression of the ETV6-NTRK3 gene fusion as a primary event in human secretory breast carcinoma. Cancer Cell.

[123] Vaishnavi A., Capelletti M., Le A.T., Kako S., Butaney M., Ercan D., Mahale S., Davies K.D., Aisner D.L., Pilling A.B. (2013). Oncogenic and drug-sensitive NTRK1 rearrangements in lung cancer. Nat. Med..

[124] Wiesner T., He J., Yelensky R., Esteve-Puig R., Botton T., Yeh I., Lipson D., Otto G., Brennan K., Murali R. (2014). Kinase fusions are frequent in Spitz tumours and spitzoid melanomas. Nat. Commun..

[125] Vaishnavi A., Le A.T., Doebele R.C. (2015). TRKing down an old oncogene in a new era of targeted therapy. Cancer Discov..

[126] Stransky N., Cerami E., Schalm S., Kim J.L., Lengauer C. (2014). The landscape of kinase fusions in cancer. Nat. Commun..

[127] Drilon A., Laetsch T.W., Kummar S., DuBois S.G., Lassen U.N., Demetri G.D., Nathenson M., Doebele R.C., Farago A.F., Pappo A.S. (2018). Efficacy of Larotrectinib in TRK Fusion-Positive Cancers in Adults and Children. N. Engl. J. Med..

[128] Xu H., Zhu X., Bao H., Wh Shek T., Huang Z., Wang Y., Wu X., Wu Y., Chang Z., Wu S. (2018). Genetic and clonal dissection of osteosarcoma progression and lung metastasis. Int. J. Cancer.

[129] Ascierto P.A., Kirkwood J.M., Grob J.J., Simeone E., Grimaldi A.M., Maio M., Palmieri G., Testori A., Marincola F.M., Mozzillo N. (2012). The role of BRAF V600 mutation in melanoma. J. Transl. Med..

[130] Nazarian R., Shi H., Wang Q., Kong X., Koya R.C., Lee H., Chen Z., Lee M.K., Attar N., Sazegar H. (2010). Melanomas acquire resistance to B-RAF(V600E) inhibition by RTK or N-RAS upregulation. Nature.

[131] Babina I.S., Turner N.C. (2017). Advances and challenges in targeting FGFR signalling in cancer. Nat. Rev. Cancer.

[132] Porta R., Borea R., Coelho A., Khan S., Araujo A., Reclusa P., Franchina T., Van Der Steen N., Van Dam P., Ferri J. (2017). FGFR a promising druggable target in cancer: Molecular biology and new drugs. Crit. Rev. Oncol. Hematol..

[133] Chae Y.K., Ranganath K., Hammerman P.S., Vaklavas C., Mohindra N., Kalyan A., Matsangou M., Costa R., Carneiro B., Villaflor V.M. (2017). Inhibition of the fibroblast growth factor receptor (FGFR) pathway: The current landscape and barriers to clinical application. Oncotarget.

[134] Zhao X., Pak E., Ornell K.J., Pazyra-Murphy M.F., MacKenzie E.L., Chadwick E.J., Ponomaryov T., Kelleher J.F., Segal R.A. (2017). A Transposon Screen Identifies Loss of Primary Cilia as a Mechanism of Resistance to SMO Inhibitors. Cancer Discov..

[135] Feddersen C.R., Schillo J.L., Varzavand A., Vaughn H.R., Wadsworth L.S., Voigt A.P., Zhu E.Y., Jennings B.M., Mullen S.A., Bobera J. (2019). Src-Dependent DBL Family Members Drive Resistance to Vemurafenib in Human Melanoma. Cancer Res..

[136] WHO Obesity and Overweight. https://www.who.int/news-room/fact-sheets/detail/obesity-and-overweight.

[137] Unger R.H., Scherer P.E. (2010). Gluttony, sloth and the metabolic syndrome: A roadmap to lipotoxicity. Trends Endocrinol. Metab..

[138] Parekh N., Chandran U., Bandera E.V. (2012). Obesity in cancer survival. Annu. Rev. Nutr..

[139] Park J., Euhus D.M., Scherer P.E. (2011). Paracrine and endocrine effects of adipose tissue on cancer development and progression. Endocr. Rev..

[140] Renehan A.G., Tyson M., Egger M., Heller R.F., Zwahlen M. (2008). Body-mass index and incidence of cancer: A systematic review and meta-analysis of prospective observational studies. Lancet.

[141] Bhaskaran K., Douglas I., Forbes H., dos-Santos-Silva I., Leon D.A., Smeeth L. (2014). Body-mass index and risk of 22 specific cancers: A population-based cohort study of 5.24 million UK adults. Lancet.

[142] Arnold M., Pandeya N., Byrnes G., Renehan P.A.G., Stevens G.A., Ezzati P.M., Ferlay J., Miranda J.J., Romieu I., Dikshit R. (2015). Global burden of cancer attributable to high body-mass index in 2012: A population-based study. Lancet Oncol..

[143] Campbell P.T., Newton C.C., Dehal A.N., Jacobs E.J., Patel A.V., Gapstur S.M. (2012). Impact of body mass index on survival after colorectal cancer diagnosis: The Cancer Prevention Study-II Nutrition Cohort. J. Clin. Oncol..

[144] Bastarrachea J., Hortobagyi G.N., Smith T.L., Kau S.W., Buzdar A.U. (1994). Obesity as an adverse prognostic factor for patients receiving adjuvant chemotherapy for breast cancer. Ann. Intern. Med..

[145] Meyerhardt J.A., Tepper J.E., Niedzwiecki D., Hollis D.R., McCollum A.D., Brady D., O’Connell M.J., Mayer R.J., Cummings B., Willett C. (2004). Impact of body mass index on outcomes and treatment-related toxicity in patients with stage II and III rectal cancer: Findings from Intergroup Trial 0114. J. Clin. Oncol..

[146] Barlow W.E., White E., Ballard-Barbash R., Vacek P.M., Titus-Ernstoff L., Carney P.A., Tice J.A., Buist D.S., Geller B.M., Rosenberg R. (2006). Prospective breast cancer risk prediction model for women undergoing screening mammography. J. Natl. Cancer Inst..

[147] Calle E.E., Rodriguez C., Walker-Thurmond K., Thun M.J. (2003). Overweight, obesity, and mortality from cancer in a prospectively studied cohort of U.S. adults. N. Engl. J. Med..

[148] Semenkovich C.F. (2006). Insulin resistance and atherosclerosis. J. Clin. Investig..

[149] Berg A.H., Scherer P.E. (2005). Adipose tissue, inflammation, and cardiovascular disease. Circ. Res..

[150] Nieman K.M., Kenny H.A., Penicka C.V., Ladanyi A., Buell-Gutbrod R., Zillhardt M.R., Romero I.L., Carey M.S., Mills G.B., Hotamisligil G.S. (2011). Adipocytes promote ovarian cancer metastasis and provide energy for rapid tumor growth. Nat. Med..

[151] Tessitore L., Vizio B., Pesola D., Cecchini F., Mussa A., Argiles J.M., Benedetto C. (2004). Adipocyte expression and circulating levels of leptin increase in both gynaecological and breast cancer patients. Int. J. Oncol..

[152] Park J., Scherer P.E. (2012). Adipocyte-derived endotrophin promotes malignant tumor progression. J. Clin. Investig..

[153] Elinav E., Nowarski R., Thaiss C.A., Hu B., Jin C., Flavell R.A. (2013). Inflammation-induced cancer: Crosstalk between tumours, immune cells and microorganisms. Nat. Rev. Cancer.

[154] O’Sullivan K.E., Reynolds J.V., O’Hanlon C., O’Sullivan J.N., Lysaght J. (2014). Could signal transducer and activator of transcription 3 be a therapeutic target in obesity-related gastrointestinal malignancy?. J. Gastrointest. Cancer.

[155] Chien Y., Scuoppo C., Wang X., Fang X., Balgley B., Bolden J.E., Premsrirut P., Luo W., Chicas A., Lee C.S. (2011). Control of the senescence-associated secretory phenotype by NF-kappaB promotes senescence and enhances chemosensitivity. Genes. Dev..

[156] Exley M.A., Hand L., O’Shea D., Lynch L. (2014). Interplay between the immune system and adipose tissue in obesity. J. Endocrinol..

[157] Weisberg S.P., McCann D., Desai M., Rosenbaum M., Leibel R.L., Ferrante A.W. (2003). Obesity is associated with macrophage accumulation in adipose tissue. J. Clin. Investig..

